# Genetics of spot blotch resistance in bread wheat (*Triticum aestivum* L.) using five models for GWAS

**DOI:** 10.3389/fpls.2022.1036064

**Published:** 2023-01-18

**Authors:** Sahadev Singh, Shailendra Singh Gaurav, Neeraj Kumar Vasistha, Uttam Kumar, Arun Kumar Joshi, Vinod Kumar Mishra, Ramesh Chand, Pushpendra Kumar Gupta

**Affiliations:** ^1^ Molecular Biology Laboratory, Department of Genetics and Plant Breeding, Chaudhary Charan Singh University, Meerut, India; ^2^ Department of Genetics-Plant Breeding and Biotechnology, Dr Khem Singh Gill, Akal College of Agriculture, Eternal University, Sirmaur, India; ^3^ Borlaug Institute for South Asia (BISA), Ludhiana, India; ^4^ The International Maize and Wheat Improvement Center (CIMMYT), Borlaug Institute for South Asia (BISA), G-2, B-Block, NASC Complex, DPS Marg, New Delhi, India; ^5^ Department of Genetics and Plant Breeding, Indian Institute of Agricultural Science, Banaras Hindu University, Varanasi, India; ^6^ Department of Mycology and Plant Pathology, Indian Institute of Agricultural Science Banaras Hindu University, Varanasi, India; ^7^ Murdoch’s Centre for Crop & Food Innovation, Murdoch University, Murdoch, WA, Australia

**Keywords:** *Triticum aestivum* L, GWAS, MTA, epistasis, candidate genes

## Abstract

Genetic architecture of resistance to spot blotch in wheat was examined using a Genome-Wide Association Study (GWAS) involving an association panel comprising 303 diverse genotypes. The association panel was evaluated at two different locations in India including Banaras Hindu University (BHU), Varanasi (Uttar Pradesh), and Borlaug Institute for South Asia (BISA), Pusa, Samastipur (Bihar) for two consecutive years (2017-2018 and 2018-2019), thus making four environments (E1, BHU 2017-18; E2, BHU 2018-19; E3, PUSA, 2017-18; E4, PUSA, 2018-19). The panel was genotyped for 12,196 SNPs based on DArT-seq (outsourced to DArT Ltd by CIMMYT); these SNPs included 5,400 SNPs, which could not be assigned to individual chromosomes and were therefore, described as unassigned by the vendor. Phenotypic data was recorded on the following three disease-related traits: (i) Area Under Disease Progress Curve (AUDPC), (ii) Incubation Period (IP), and (iii) Lesion Number (LN). GWAS was conducted using each of five different models, which included two single-locus models (CMLM and SUPER) and three multi-locus models (MLMM, FarmCPU, and BLINK). This exercise gave 306 MTAs, but only 89 MTAs (33 for AUDPC, 30 for IP and 26 for LN) including a solitary MTA detected using all the five models and 88 identified using four of the five models (barring SUPER) were considered to be important. These were used for further analysis, which included identification of candidate genes (CGs) and their annotation. A majority of these MTAs were novel. Only 70 of the 89 MTAs were assigned to individual chromosomes; the remaining 19 MTAs belonged to unassigned SNPs, for which chromosomes were not known. Seven MTAs were selected on the basis of minimum P value, number of models, number of environments and location on chromosomes with respect to QTLs reported earlier. These 7 MTAs, which included five main effect MTAs and two for epistatic interactions, were considered to be important for marker-assisted selection (MAS). The present study thus improved our understanding of the genetics of resistance against spot blotch in wheat and provided seven MTAs, which may be used for MAS after due validation.

## Introduction

Wheat is the third most important crop world-wide, next only to rice and maize, contributing ~20% of total dietary calories and proteins worldwide (Shiferaw et al., 2013). Although the global production of wheat has been able to keep pace with the demand and consumption during the last more than five decades, the decline in rate of annual production from 3% in the past during green revolution to <1% in recent years has been a cause of alarm, since 18% increase in global wheat production will be needed by the year 2050, according to some available estimates (Alexandratos and Bruinsma, 2012). Therefore, there is a need to improve the productivity and production of wheat to meet future demands. In this connection, it is relevant to recognize that the productivity of wheat has been constantly under threat due to a variety of biotic and abiotic stresses. Among biotic stresses, several diseases cause major yield losses to wheat crop everywhere in the world. Spot blotch has its own share in this loss in productivity. According to some estimates, globally spot blotch affects >25 million ha, representing 12% of the total wheat area (Duveiller et al., 2005). Geographically, this affected area includes parts of South Asia (including North-Eastern Plain Zone of India, Bangladesh, and Tarai Region of Nepal), South-East Asia (including Thailand, Philippines, Indonesia, and China), Latin America (including North East part of Argentina, Bolivia, warmest area of Brazil, Paraguay) and Africa (including Tanzania and Zambia) (Joshi et al., 2002; Joshi et al., 2007b; Chatrath et al., 2007; Juliana et al., 2022). According to different estimates, yield losses due to SB, in different years, ranged from 0% to 100% in different parts of the world (Sharma and Duveiller, 2006; Siddique et al., 2006; Juliana et al., 2022); complete destruction of the crop leading to 100% loss occurs only under conditions, favourable for the pathogen, (Mehta, 1985; Saari, 1998). The end-use quality of harvested grain is also affected, since the pathogen infects the spikes and the grain (Singh et al., 2015; Gupta et al., 2018a; Gupta et al., 2018b; Kumar et al., 2019).

It is widely known that the use of resistant cultivars is the safest means to safeguard against yield losses and is also environmentally safe. A knowledge of the genetics of resistance in the host is necessary for the development of these resistant cultivars. Therefore, a large number of studies for the study of the genetics of resistance have been undertaken in the past. The early inheritance studies suggested monogenic to polygenic resistance with the involvement of dominant as well as recessive genes. The availability of dominant as well as recessive nature of disease resistance in these different studies has been attributed to the use of parents with different genetic constitutions and the possibility of same genes behaving as dominant in one genetic background and recessive in the other (for a review, see Gupta et al., 2018a).

For identification of the genes involved in resistance to SB in wheat, the trait has been treated as a qualitative trait in some studies and as a quantitative trait in some other relatively recent studies. While treating it as a quantitative trait, QTL analysis involved either interval mapping or genome-wide association studies (GWAS) (Kumar et al., 2009; Kumar et al., 2010; Marone et al., 2013; French et al., 2016; Juliana et al., 2022). As a qualitative trait, two different classes of genes were identified, the first having four Sb genes, which follow a gene-for-gene (GFG) relationship (Flor, 1955), and the second with a solitary sensitivity gene *Tsn1*, which follows an inverse gene-for-gene relationship (IGFG). The four Sb genes in the former category included the following: *Sb1* (7DS), *Sb2* (5BL), *Sb3* (3BS) and *Sb4* (4BL) (Lillemo et al., 2013; Kumar et al., 2015; Lu et al., 2016; Zhang et al., 2020), but no corresponding avirulence (Avr) genes is known for any of these four genes. But for sensitivity gene *Tsn1* in the second category, the corresponding virulence gene *ToxA* is known (Navathe et al., 2020).

A number of interval mapping studies for the identification of QTLs and GWA studies for identification of MTAs have also been conducted (for a review, see Gupta et al., 2018a). These QTLs and MTAs are listed in “WheatQTLdatabase” that was recently developed at our centre (Singh et al., 2021; Singh et al., 2022). The most comprehensive recent GWA study, however, involved six association panels from a large set of 6,736 advanced bread wheat breeding lines from the International Maize and Wheat Improvement Center (CIMMYT) (Juliana et al., 2022). A meta-QTL analysis involving QTLs for resistance against spot blotch and other related diseases has also been conducted at our centre, leading to the identification of 30 M-QTLs based on 87 of the 241 QTLs identified so far using interval mapping (our unpublished results).

The pathogen (*B. sorokiniana*) has also been studied for occurrence of races/pathotypes and identification of genes involved in pathogenesis. In several studies, isolates have been collected from specific geographical regions and differentials suggested for classification of these isolates into groups, sometimes erroneously described as pathotypes (Aggarwal et al., 2009; Chauhan et al., 2017; also see reviews by Gupta et al., 2018a and Navathe et al., 2022). So far, no physiological races or pathotypes have been identified and described, although a virulence gene has been identified (*VHv1* in barley and *VTa1* in wheat; Zhong et al., 2002). However, no avirulence (Avr) gene for any Sb gene following GFG model and/or effector molecule derived from the pathogen (*B. sorokinaia*) has been identified. However, a virulence gene *ToxA* in the pathogen and its sensitivity gene *Tsn1* in the host following IGFG for spot blotch was identified recently (McDonald et al., 2018), although this pair of genes for other necrotrophic diseases like Septoria blotch and tan spot was known for quite some time (Gupta et al., 2022 for a review). Prevalence of *ToxA* in wheat genotypes and that of *Tsn1* in pathogen isolates has also been examined. For instance, Navathe et al. (2020) reported occurrence of *ToxA* gene in 70% isolates (77 of 110 Indian isolates) and *Tsn1* gene in 36.8% of wheat genotypes (81 of 220 wheat genotypes). Whole genome sequence of the pathogen (*B. sorokiniana*) has also been worked out recently and putative avirulence genes suggested, but these genes need to be validated through further investigations (Aggarwal et al., 2022).

Despite the progress outlined above, we feel convinced that the available genes/QTLs/MTAs do not represent the entire repertoire of gene loci or QTLs that may be involved in providing resistance against spot blotch, and that there is a scope for finding additional novel MTAs using additional germplasm. Keeping this in view, a GWA study was undertaken using five different models to identify novel marker trait association (MTAs) and to detect loci which provide spot blotch resistance through a novel association panel (never used earlier). As expected, many novel MTAs involved in spot blotch resistance were identified in the present study. The results of the study are presented and discussed in this communication.

## Material and methods

### Association panel and experimental design

The GWAS panel consisted of 303 diverse wheat accessions (a set of Spring Wheat Reference Set also known as SWRS population which procured from CIMMYT gene bank, Mexico; for details of 303 genotypes, see **Supplementary Table 1**) and was genotyped for 12,160 (with MAF of <5%) of the 17, 937 SNPs that were generated using NGS-based DArT-seq using Illumina platform under the “Seed for Discovery” project of CIMMYT Mexico (outsourced by CIMMYT to Diversity Array Technology Pvt. Ltd. Australia). Of these 12,160 SNPs, 5,400 SNPs were described as unassigned, since these SNPs could not be assigned to specific chromosomes.

Following alignment, filtering was applied in order to detect the best assignment/anchorage to a physical position on the reference genome using the default criteria of Bowtie 2: The following criteria were used for filtering: (i) unique mapping to an unambiguous locus; (ii) maximum 1 bp mismatch to the marker sequence, and (iii) markers with multiple alignment options discarded, if the second-best hit showed < 3 bp mismatch to the marker sequence, i.e. markers with 2 or more hits (loci) were discarded if there was not at least 3 bp difference between the best and second-best hit. (iv) Monomorphic markers were discarded as well as (v) SNPs with MAF (Minor Allele Frequency less than 5% and more than 5% missing data).

The panel was raised in a CRBD with two replications in each of the following four environments: E1 (2017-2018) and E2 (2018-2019) at Agriculture Research Farm, BHU, Varanasi, UP, E3 (2017-2018) and E4 (2018-2019) at BISA Agriculture farm, Pusa, Samastipur, Bihar. Recommended crop management practices were followed (i.e., 200 kg/ha fertilizer; N: P: K = 8: 8: 8). Each genotype in a replication was represented by a plot of 3 rows of 1m each, with a row-to-row distance of 0.25 m.

### Inoculation and recording of phenotypic data

Pure culture of a highly aggressing isolate of the pathogen (HD 3069, BHU, Varanasi, India) was multiplied on sorghum grain and used for inoculation following Chaurasia et al. (1999). Development of spot blotch was ensured through use of agronomic practices (including frequent irrigations) to create environment conducive for the pathogen. Phenotypic data were recorded on the following three disease related traits: (i) Area Under Disease Progress Curve (AUDPC; 00-99, double digit data). (ii) Incubation Period (IP; in days). (iii) Lesion number (LN).

For AUDPC, disease severity (%) was recorded in three different growth stages (GS), GS 63 (beginning of anthesis to half complete), GS 69 (anthesis complete) and GS 77 (late milking). Disease severity was assessed by the formula [(1/9 (D1×D2) ×100] using the double-digit scale (DD, 00–99) (Saari and Prescott, 1975). The first digit (D1) refers to vertical disease progress on the plant, whereas the second digit (D2) was the disease severity score in the affected leaves. Thus, disease severity was used to estimate the AUDPC by following formula (Sharma and Duveiller, 2007).


$$AUDPC\;=\sum \;1/2\times \left(Y_{i}+\;Y_{\left(i+1\right)}\right)*\left(t_{\left(i+1\right)}–\;t_{i}\right)$$

Where Y_i_ and Y _(i + 1)_ = disease severity at time t_i_ and t _(i + 1)_, respectively; t _(i + 1)_—t _i_ = time interval (number of days) between two disease scores assessed.

IP was recorded as the duration (in number of days) from inoculation to the appearance of visible symptoms on five randomly tagged plants in each plot (Parlevliet, 1979). Similarly, for LN, five random flag leaves were each divided into four parts with a marker pen and the number of lesions on each part was counted. The number of spots from each part were added and the total number of spots was used as LN (Roumen, 1992).

### Statistical analysis and frequency distribution

ANOVA and correlation coefficients were obtained using Agricolae package in R studio. Violin plots for phenotypic data were developed for each of the four individual environments and BLUP values. For this purpose, BLUP values were obtained using the ‘lme4’ in R (Bates et al., 2015). Descriptive statistics including mean, standard deviation, coefficient of variation (CV) was obtained using SPSS v. 17.0 (SPSS Inc 2008).

### Principal components, population structure and kinship matrix

Genotype data were available for 17,937 SNPs, but only 12,160 markers remained after filtering out those with a marker allele frequency (MAF) of less than 5%. These 12,160 SNPs were then employed in PCA/population structure analysis and GWAS.

The principal component (PC) analysis using genotypic data was conducted for the development of population structure (Q matrix) and relatedness (K matrix) using tools available in GAPIT (Lipka et al., 2012); Q and K matrices were obtained using default set of parameters (VanRaden, 2008; Lipka et al., 2012). The first three PCs were used to produce a 3D scatter plot showing distribution of genotypes into sub-groups.

Population structure involving 210 SNPs (ten SNPs from each chromosome) was examined using the software STRUCTURE version 2.3.4 (Pritchard et al., 2000). The details of the procedure followed are available in an earlier publication from our lab, where the same association panel with minor differences was employed (Kumar et al., 2018).

### Identification of marker trait associations

Following five different models were used for GWAS: (i) Compressed mixed linear model (CMLM; Zhang et al., 2010); (ii) Settlement of MLM Under Progressively Exclusive Relationship (SUPER; Wang et al., 2014); (iii) Multi locus mixed-model (MLMM; Segura et al., 2012); (iv) Fixed and random model Circulating Probability Unification (FarmCPU; Liu et al., 2016); (v) Bayesian-information and Linkage-disequilibrium Iteratively Nested Keyway (BLINK; Huang et al., 2018). The first two models are single locus models and the remaining three are multi-locus models. These models are freely available in Latest version of Genomic Association and Prediction Integrated Tool (GAPIT V.3) (Wang and Zhang, 2021). Default significance threshold value implemented by GAPIT was FDR<0.05. But since the FDR implemented in GAPIT seems to be very stringent/conservative, no significant MTAs could be identified for any of the traits; therefore, GWAS using P<0.001 was also conducted, as also done earlier in wheat (Wang et al., 2017) and other cereals including rice (Feng et al., 2016). Significant MTAs were identified at a stringent probability value of P<0.001 and MTAs were represented in the form of maps using MAPCHART software (Voorrips, 2002).

### Epistasis analysis (SNP×SNP interaction)

PLINK provides several alternatives for selecting the pairs of SNPs to be used for epistatic interactions, which means either we can use all available pairs of SNPs or select only a limited number of pairs, using any one of the several criteria provided. In the present study, epistatic interactions were identified using all the possible pairs of SNPs (12,160 SNPs give 73,926,720 pairs) was carried out by using PLINK^2^ (Purcell et al., 2007). It is freely available and command-based package of tools for whole genome association analysis. Significant interactions were filtered at p-value <1×10^-8^ (Purcell et al., 2007; Jan et al., 2019). The SNPs involved in epistatic interactions were described as E-QTNs.

### Identification of putative candidate genes

The most significant MTAs detected were also used for identification of CGs through alignment of sequences associated with MTAs with wheat genome assembly IWGSC v.1 (International Wheat Genome Sequencing Consortium (IWGSC) et al., 2018) that is hosted on the Ensembl database^3^. Highly significant annotated CGs were retrieved from a 200 kb window for each MTA. IWGSC^4^ was used for gene ontology (GO) annotation information of these CGs.

## Results

**Figure 1:**
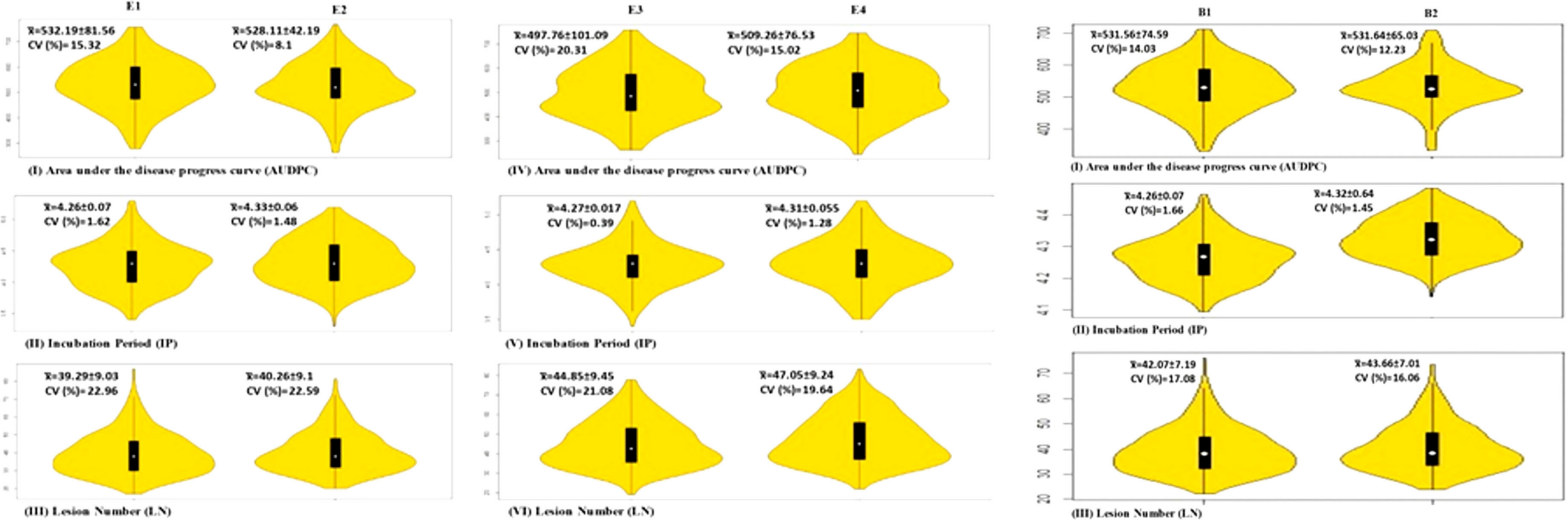
Violin plots for three traits in four environments and BLUP; means are shown by vertical solid black bars with means shown as white dots within the bars.

Violin plots showing variation for three spot blotch traits in four individual environments and BLUP are depicted in **Figure 1**. The results of the analysis of variance (ANOVA) are presented in **Table 1**.

**Table 1:** ANOVA for three traits: (i) area under disease progress curve (AUDPC), (ii) incubation period (IP) and (iii) lesion number (LN).

Source of variation	Df	Mean sum of squares		
		AUDPC	IP	LN
Env	1	430117*	0.0	23144*
Year	1	8332	1.3*	1524*
Genotype	302	36805*	0.3*	735*
Rep	1	7.6	1.4*	30.5
Env × Genotype	302	35972*	0.3*	342*
Year × Genotype	302	2817	0.1	11.5

*Significant at the p-value (P<0.01) probability level; Df, degrees of freedom.

### Principal components analysis and population structure

**Figure 2:**
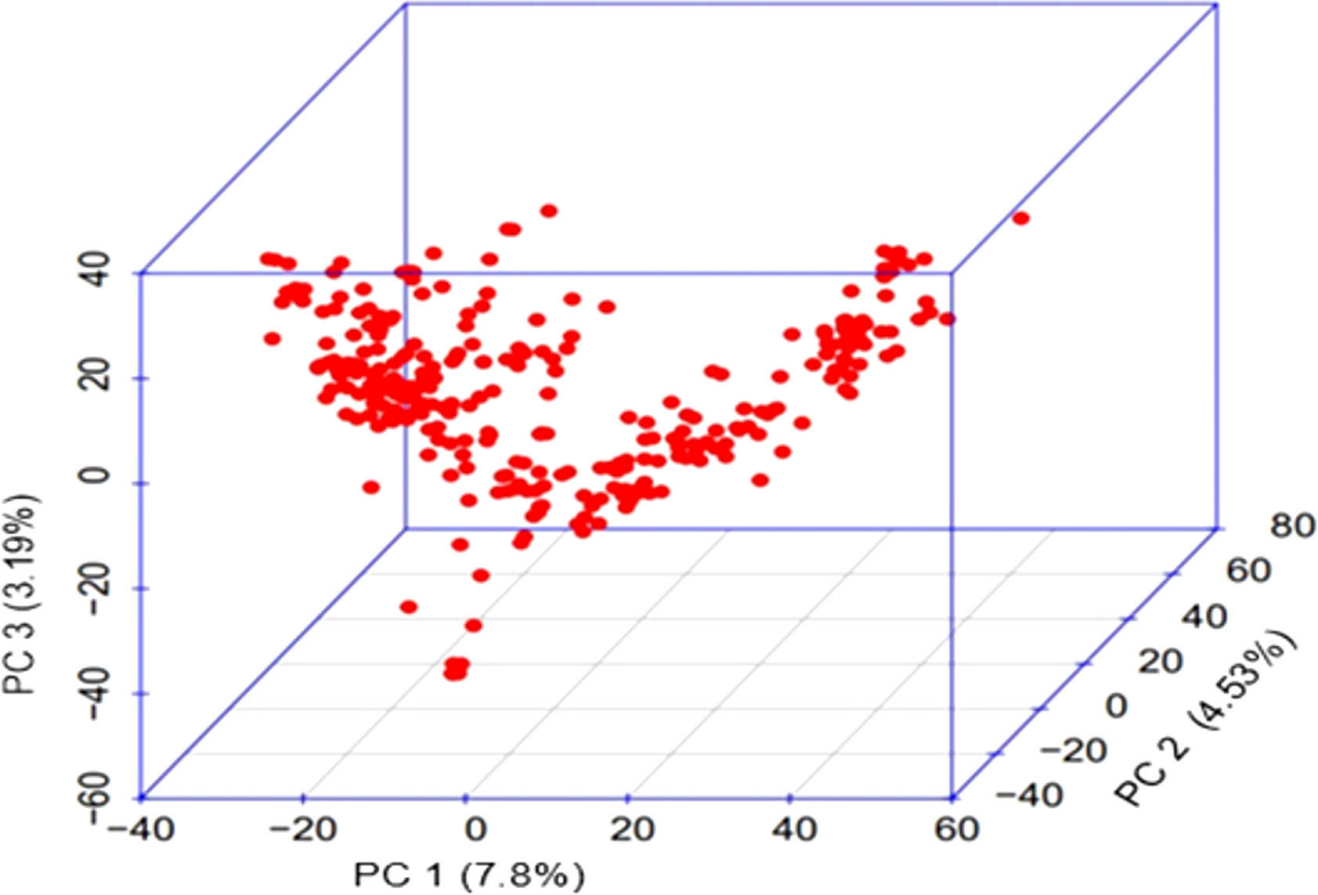
3D PC scatter plot (3D PC) for genotypes based on the first three principal components (PC1, PC2 and PC3).

**Figure 3:**
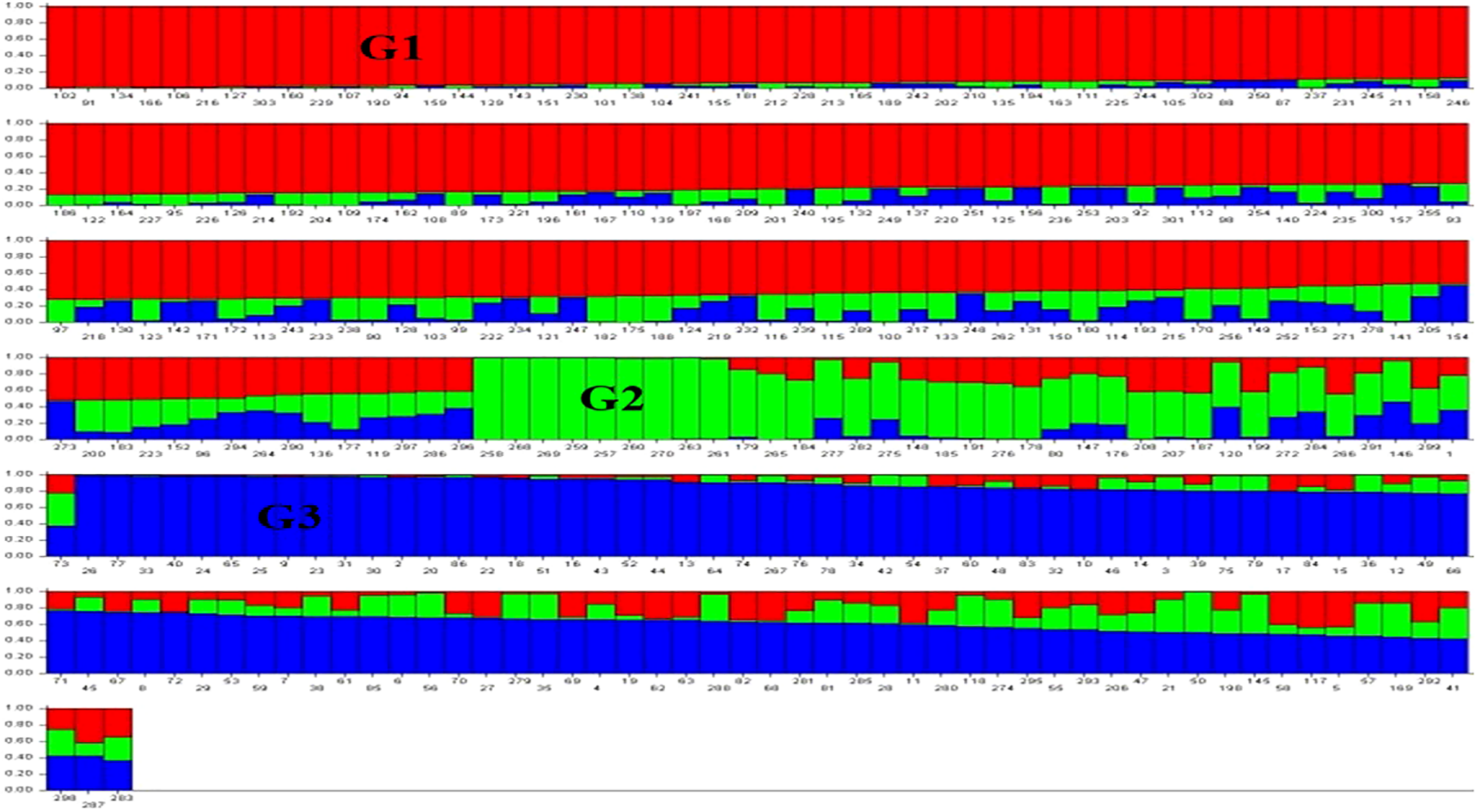
Population structure showing three sub-groups (G1, G2, G3) by three different colours (red, green, and blue); the subgroup IV representing admixture had genotypes with more than one color. Each bar represents one genotype.

The results for the first three PCs (PC1, PC2 and PC3) are presented in **Figure 2**, suggesting the presence of three sub-groups in the association panel. Following population structure analysis using 210 unlinked markers, 303 genotypes were placed in four subgroups, with 75 genotypes in subgroup I, 11 genotypes in subgroup II, 42 in subgroup III and the remaining 175 in the admixture group IV (**Figure 3**). The information generated by population structure was used for developing Q matrix for GWAS.

### GWAS and marker trait associations

**Figure 4:**
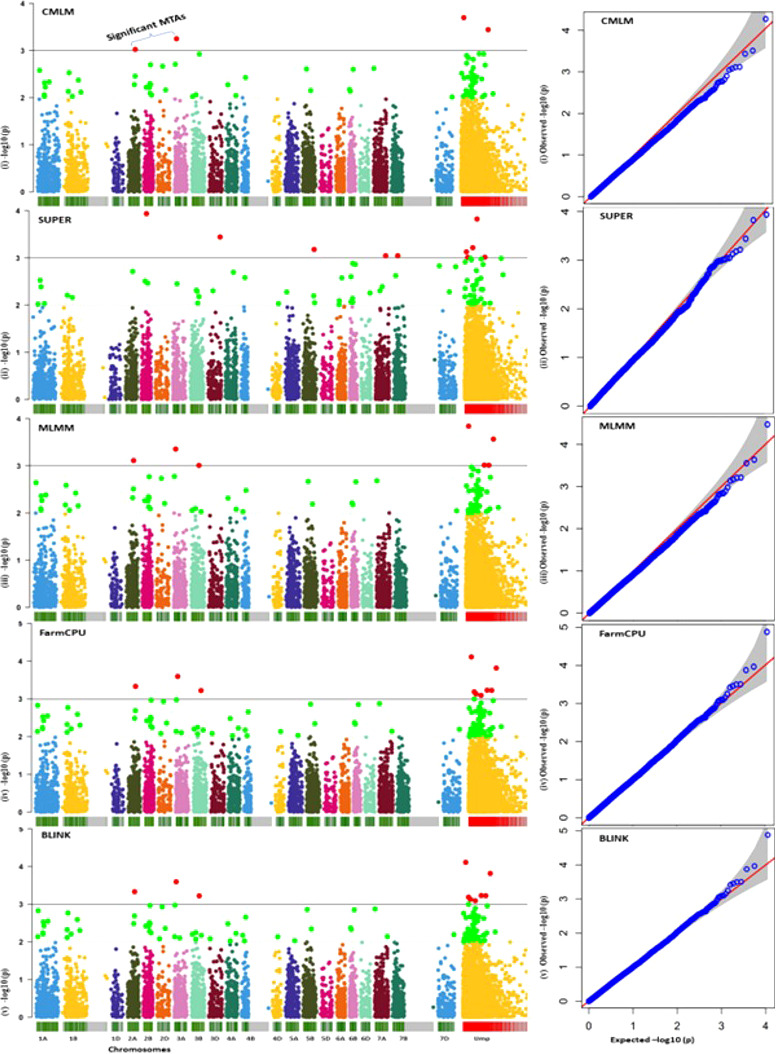
A set of representative Manhattan plots (left panel) and Q–Q plots (right panel; expected values shown as red line assuming no association) for five models (for only BLUP values for AUDPC); red dots above the horizontal line depict significant MTAs.

**Figure 5:**
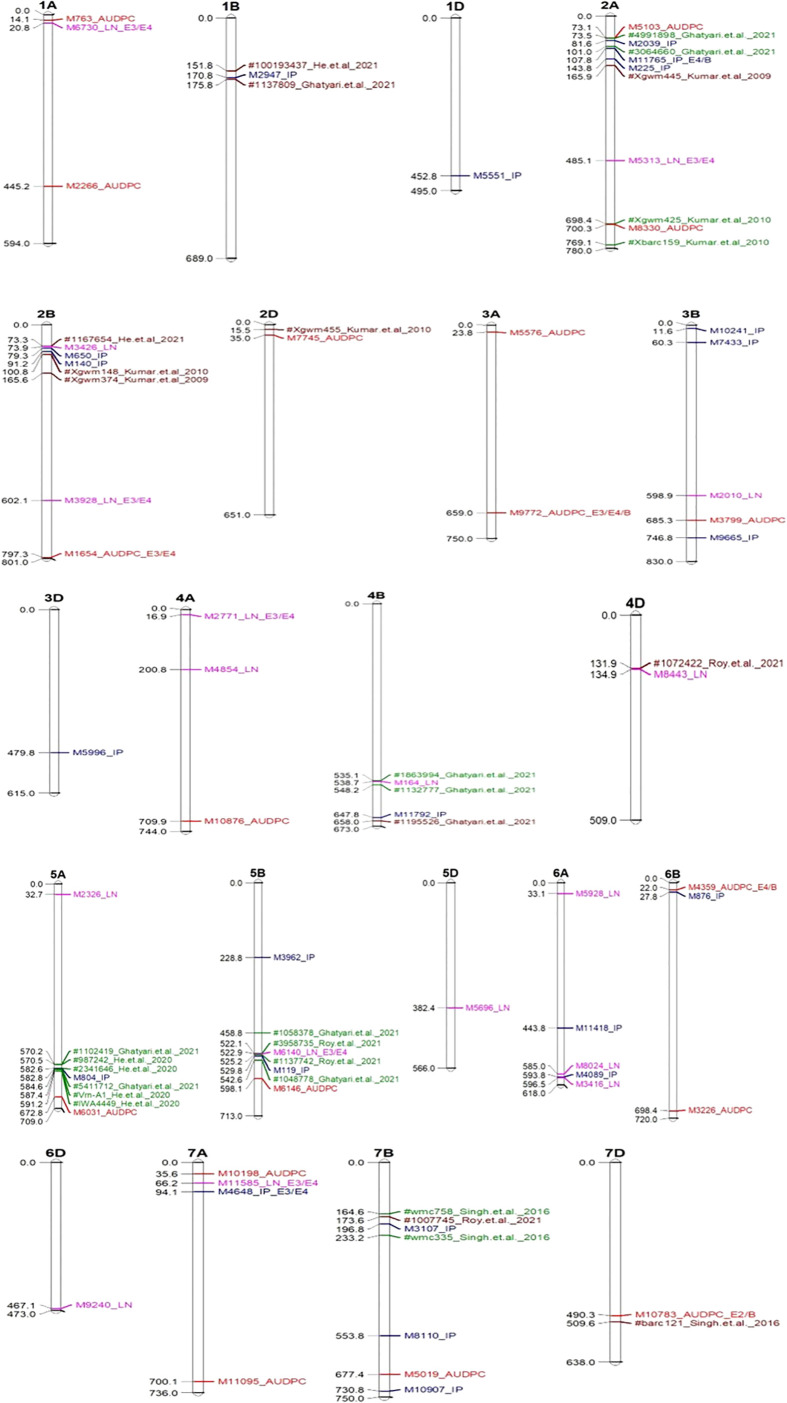
Significant MTAs associated with QTLs reported earlier (Green and Brown colour) mapped on different chromosome. In the above figures significant MTAs for AUDPC indicated by red colour; IP by blue colour & LN by pink colour; E1-Enivironment 1; E2-Enivironment 2; E3-Enivironment 3; E4-Enivironment 4; B-BLUP.

A representative set of Manhattan plots and QQ plots based on BLUP data are depicted in **Figure 4**. Manhattan plots for all 60 combinations (3 traits, 4 environments and 5 models) are available in **Supplementary Figures 1–12**. The total number of MTAs involving three traits and five models were 306 (91 for AUDPC; 100 for IP and 115 for LN; **Supplementary Tables 2–4**). A summary of 89 MTAs including one MTA found in all the five models and 88 that were common among four models (except SUPER) is presented in **Table 2** and **Figure 5**. These 89 MTAs include 33 for AUDPC, 30 for IP and 26 for LN. Among these MTAs, 12 MTAs occurred in more than one environment (four for AUDPC, two for IP, and six for LN) and 19 MTAs belongs to the category of unassigned SNPs. Assignment of remained 57 MTAs; 18 for AUPDC (located on 13 chromosomes), 22 for IP (located on 13 chromosomes), 17 for LN (located on 13 chromosomes) on individual chromosomes is shown in **Figure 5**.

**Table 2:** A Summary of MTAs for each trait (one MTA in bold was detected by all the five models; the remaining MTAs were detected by four models except SUPER; NA=not available).

SNP	Chr.	Pos. of SNP tag (bp)	-log10 (P)	maf	SNP	Chr.	Pos. of SNP tag (bp)	-log10(P)	maf
AUDPC (E1:7; E2:4; E3:4; E4:7 and BLUP:11)									
E1									
M2266: A/G	1A	445269309-445269377	8.37E-04	0.445545	M1654: C/A	2B	797177741-797377809	2.36E-04	0.237624
M3799: T/A	3B	685318543-685318611	3.68E-04	0.065677	M4359: A/T	6B	22081357-22081425	6.66E-04	0.356304
M5103: G/C	2A	73179815-73179883	8.98E-04	0.227723	M8026: A/G	NA	NA	4.53E-04	0.236584
M6031: C/G	5A	672843173-672843241	5.36E-05	0.095215	M9772: A/G	3A	659038152-659038220	2.32E-04	0.285462
M7254: G/A	NA	NA	3.09E-04	0.11495	M10198: T/G	7A	35630453-35630518	6.17E-04	0.057574
M10592: G/C	NA	NA	7.72E-04	0.062706	M11870: C/T	NA	NA	2.70E-04	0.156617
M10876: G/A	4A	709908601-709908669	7.63E-04	0.235066	B				
E2					M763: C/G	1A	14161093-14161161	4.38E-04	0.462046
M5576: T/C	3A	23840923-23840991	5.63E-04	0.206254	M3226: T/C	6B	698461241-698461309	5.85E-04	0.321634
M6146: A/C	5B	598120554-598120622	3.61E-04	0.360264	M4359: A/T	6B	22081357-22081425	4.66E-04	0.356304
M8330: A/C	2A	700374249-700374317	9.51E-04	0.155116	M5019: C/T	7B	677430865-677430933	4.18E-04	0.326337
M10783: G/A	7D	490344136-490344204	2.01E-04	0.223531	M7025: T/C	NA	NA	7.40E-04	0.055941
E3					M7745: G/C	2D	35039085-35039153	2.89E-04	0.431749
M976: G/C	NA	NA	3.36E-04	0.257228	M9397: C/A	NA	NA	5.26E-04	0.116386
M1654: C/A	2B	797177741-797377809	4.09E-04	0.237624	M9772: A/G	3A	659038152-659038220	1.25E-04	0.285462
M8026: A/G	NA	NA	4.99E-04	0.236584	M10783: G/A	7D	490344136-490344204	1.82E-04	0.223531
M9772: A/G	3A	659038152-659038220	1.16E-04	0.285462	M11095: A/G	7A	700115446-700115514	7.88E-04	0.079076
E4					M11338: T/C	NA	NA	9.06E-04	0.459703
M976: G/C	NA	NA	5.54E-05	0.257228					
Incubation Period (E1:3; E2:2; E3:12; E4:6 and BLUP:7)									
E1									
M140: A/C	2B	91205274-91205342	4.95E-04	0.158416	M9264: G/C	NA	NA	7.62E-04	0.174719
M650: C/A	2B	79317077-79317117	4.37E-04	0.19802	M10907: G/T	7B	730875968-730876036	1.24E-04	0.278696
M11792: C/G	4B	647704512-647704580	2.46E-04	0.348878	E4				
E2					M4648: G/T	7A	94143005-94143073	2.78E-04	0.285132
M8531: T/C	NA	NA	9.08E-04	0.108812	M5996: A/C	3D	479617743-479617811	9.84E-04	0.370875
M11418: G/A	6A	443334314-443334382	8.30E-04	0.203663	M7433: T/C	3B	60372653-60372721	3.22E-04	0.17495
E3					M10241: G/T	3B	11646443-11646511	2.24E-04	0.051139
M119: A/G	5B	529608885-529608950	5.53E-04	0.064274	M11765: T/C	2A	107180183-107180251	3.43E-04	0.155116
M225: A/G	2A	143209405-143209473	8.46E-04	0.432343	M12439: G/C	NA	NA	8.90E-04	0.183828
M804: C/T	5A	582958870-582958938	3.90E-04	0.054406	B				
M3107: T/C	7B	196255937-196256005	5.85E-04	0.05264	**M876: G/C**	6B	27649755-27649823	8.16E-04	0.498548
M3962: C/T	5B	228277790-228277858	6.15E-04	0.318399	M2039: C/G	2A	81660284-81660352	1.32E-04	0.062706
M4648: G/T	7A	94143005-94143073	5.89E-05	0.285132	M2947: T/G	1B	170842706-170842774	7.09E-04	0.395776
M4837: T/C	NA	NA	1.86E-04	0.224422	M4089: T/A	6A	593657698-593657766	1.81E-04	0.159719
M4958: G/A	NA	NA	9.23E-04	0.113449	M7868: T/C	NA	NA	3.70E-04	0.156155
M5551: T/G	1D	452208385-452208453	8.56E-04	0.256997	M9665: C/T	3B	746854760-746854828	9.63E-04	0.154422
M8110: T/C	7B	553167382-553167450	7.43E-04	0.074868	M11765: T/C	2A	107180183-107180251	3.43E-04	0.155116
Lesion Number (E1:6; E2:4; E3:9; E4:6 and BLUP:1)									
E1									
M2326: T/C	5A	32743559-32743627	5.00E-04	0.235924	M6140: T/C	5B	522912127-522912195	3.24E-04	0.221023
M3426: A/G	2B	73990358-73990426	1.85E-04	0.480182	M6730: T/C	1A	20893697-20893765	5.11E-04	0.094604
M5696: T/G	5D	382494021-382494089	6.70E-04	0.287228	M9263: G/C	NA	NA	7.11E-04	0.440545
M8024: A/G	6A	585062562-585062630	8.90E-04	0.168944	M11585: G/A	7A	66298367-66298435	5.01E-04	0.17066
M8443: G/A	4D	134916786-134916854	3.98E-06	0.124752	M4854: T/G	4A	4A:200817628-200817696	8.44E-04	0.186056
M9994: G/C	NA	NA	4.57E-04	0.306931	M2010: T/C	3B	598995436-598995504	9.93E-04	0.359736
E2					E4				
M164: A/G	4B	538706787-538706855	5.74E-04	0.067607	M2771: A/C	4A	16997957-16998025	1.18E-04	0.308531
M992: C/T	NA	NA	7.62E-05	0.145017	M3928: G/C	2B	602182720-602182788	2.83E-04	0.438614
M3416: G/A	6A	596590906-596590974	6.52E-04	0.409373	M5313: T/C	2A	485122901-485122967	6.21E-04	0.135314
M9240: G/A	6D	467151830-467151898	2.54E-04	0.192327	M6140: T/C	5B	522912127-522912195	5.34E-04	0.221023
E3					M6730: T/C	1A	20893697-20893765	3.99E-04	0.094604
M2771: A/C	4A	16997957-16998025	6.62E-04	0.308531	M11585: G/A	7A	66298367-66298435	5.37E-04	0.17066
M3928: G/C	2B	602182720-602182788	2.12E-04	0.438614	B				
M5313: T/C	2A	485122901-485122967	4.94E-04	0.135314	M5928: T/C	6A	33188097-33188165	5.94E-04	0.250957

### Epistatic interactions

MTAs representing 44 pairs of first-order epistatic interactions (SNP × SNP interactions) are listed in **Supplementary Table 5**. Eight major interactions (two for AUDPC, one for IP and five for LN) are listed in **Table 3**.

**Table 3:** Epistatic interactions for different traits in three environments.

SNP1; SNP Allele	Ch: Pos. of SNP Tag	SNP2; SNP Allele	Ch: Pos. of SNP Tag	p- Value
AUDPC				
M238; A>G	2B: 67247748-67247765	M1164; A>C	7A: 94495522-94495590	1.27E-03
M4326; C>G	7A: 640669196-640669264	M11844; T>C	3B: 581263393-581263461	1.07E-03
IP				
M3711; G>C	1B: 499899181-499899249	M4228; T>C	7B: 572536722-572536790	4.02E-03
LN				
M921; G>A	3A: 509934375-509934443	M8709; G>A	6B: 48479624-48479692	1.57E-03
M1998; C>T	3A:12868494-12868562	M8278; G>C	6A: 606427410-606427478	7.26E-03
M7467; C>G	3D: 556059162-556059230	M12122; C>G	7D: 3854256-3854324	9.14E-03
M9322; G>T	2A: 16016622-16016690	M3401; G>A	7A: 27478985-27479053	7.55E-03
M9496; G>A	3A: 17528445-17528513	M9267; T>C	3B: 683764839-683764907	2.00E-03

Ch, Chromosome; Pos, position.

### MTAs overlapping or occurring in the vicinity of known QTLs/MTAs

When compared with 84 known QTLs reported in earlier studies, only seven MTAs of the 70 (remaining 19 were unassigned) occurred within the QTL interval (in green colour) (one associated with AUDPC, four associated with IP and two associated with LN) and the other 12 occurred in the vicinity of the markers flanking (range from 0.4 to 22.7 Mb; in brown colour; three for AUDPC, seven for IP, and two for LN) the QTLs reported earlier (**Supplementary Tables 6** and **Figure 5**); remained 38 MTAs were novel.

### Candidate genes

Genomic regions within a window of 200 kb of each MTA (100 kb on each side), when subjected to identification of CGs, gave 163 CGs (61 for AUDPC, 54 for IP and 48 for LN). These CGs were associated with only 72 MTAs (AUDPC: 26; IP: 28; and LN: 18); the remaining 21 MTAs gave no CGs. These CGs, when screened for the identification of genes already known to be involved in different pathways of pathogen–host interactions and pathogenesis, gave 64 CGs, which included 25 CGs for AUDPC, 23 for IP and 16 for LN (**Supplementary Table 7**). These CGs encoded the following 14 major proteins that are relevant to pathogenesis and pathogen–host interactions: (i) NBS-LRR domain superfamily; (ii) F-box domain superfamily; (iii) Kinase-like domain superfamily; (iv) DEAD/DEAH box helicase domain; (v) P-loop containing nucleoside triphosphate hydrolase; (vi) Senescence-associated family protein; (vii) Zinc finger like domain; (viii) Transcription factor GRAS; (ix) Helix-loop-helix DNA-binding domain superfamily; (x) Basic-leucine zipper domain; (xi) DPBB domain; (xii) Transcription factor, MADS-box superfamily; (xiii) Cytochrome P450 superfamily and (xiv) GDSL lipase/esterase-like, plant SGNH hydrolase superfamily. CGs encodes proteins which are directly or indirectly involved in host-pathogen response and are the targets for future functional genomics research focus to understand the significance of these CGs for resistance to spot blotch.

## Discussion

In the present study, an association panel comprising global collection of 303 diverse genotypes (procured from CIMMYT, Mexico) was evaluated for variation in spot blotch resistance at two different locations of India, which represented regions with warm humid climate, suitable for the spot blotch disease. The study allowed identification of genomic regions carrying markers associated with functional loci for SB resistance. High level of variability (as revealed by descriptive statistics) for each of the three traits suggested that the panel was suitable for a study of the genetics of quantitative traits. The same panel was earlier utilized by us in GWAS for several other traits including the following: (i) yield related traits (Sehgal et al., 2017; Malik et al., 2021a; Malik et al., 2021b; Malik et al., 2022); (ii) Fe, Zn, β-carotene, GPC content (Kumar et al., 2018); and, (iii) drought tolerance (Gahlaut et al., 2019).

The genotyping data for 210 SNP markers (distributed on 21 chromosomes) suggested a low level of population structure in the association panel (**Figure 2**), which is a desirable feature for GWAS, as also shown in our earlier studies involving the same association panel with minor differences (Kumar et al., 2018; Gahlaut et al., 2019; Malik et al., 2021a; Malik et al., 2021b; Malik et al., 2022). In earlier studies involving different association panels also, the number of sub-populations ranged from three (for example, Wang et al., 2017; Rahimi et al., 2019) to six (for example, Li et al., 2016; Qaseem et al., 2018; Jamil et al., 2019), suggesting that in majority of studies in wheat, the level of population structure is low. It has been shown that population structure and relatedness due to ancestry are two important confounding factors in GWAS, which were initially addressed in mixed linear model, MLM (Yu et al., 2006). In this model, the problem of population structure and relatedness were addressed though development and use of Q and K matrices (Sui et al., 2018). However, MLM had several weaknesses including computational demand, multiple testing, and background effect. Therefore, during the last 15 years, about a dozen models were proposed to address these problems. Five of these improved models were used in the present study to evaluate their relative merits.

In the present study, 306 MTAs were identified using one or more of the five models used in the present study. There was a solitary MTA (M876, present on chromosome 6), which was detected by all the five models. The remaining 305 MTAs were first placed in two groups, those identified using SUPER and those common to all the remaining four models. The MTAs other than 88 MTAs common to four models, were then classified into MTAs common to three models, two models and those unique to individual models (see **Supplementary Tables 2–4**).

One of the major issues in GWAS, is the control of both false positives as well as false negatives. The false positives are the result of occurrence of LD due to reasons other than linkage, including selection, genetic drift, etc. To control these false positives, use of Bonferroni correction and FDR were recommended. However, it has been repeatedly reported that these measures, although control false positives, but lead to false negatives, which is equally undesirable (Narum, 2006; Kaler and Purcell, 2019; White et al., 2019; Kaler et al., 2020). For the identification of significant MTAs, stringent criteria (p-value <0.001) were used. Bonferroni correction was also used in the form of built-in facility in FarmCPU and BLINK. The 88 MTAs highlighted in the present study represented those, which were detected using four of the five models (excluding SUPER), such that these MTAs do not suffer from any weaknesses of the two single locus models CMLM, SUPER and the multi-locus model MLMM. These were all identified by FarmCPU and BLINK, thus suggesting that all MTAs were valid even after Bonferroni correction. Otherwise, Bonferroni correction is widely known to give many undesirable false negatives.

The above results can be examined in light of the results of QTL analysis studies conducted (including interval mapping and GWAS) in the last three decades. A variety of molecular markers and statistical tools were utilized for the earlier studies involving all major crops. At least >30,000 QTLs for different traits including yield and tolerance to biotic and abiotic stresses are already available in wheat (for wheat QTL database, see Singh et al., 2021; Singh et al., 2022). Of these ~600 QTLs/MTAs (84 QTLs using interval mapping + 516 MTAs using GWAS) were available for resistance against spot blotch and related diseases.

Among linkage-based interval mapping studies, eleven studies were conducted for spot blotch (Kumar et al., 2009; Kumar et al., 2010; Lillemo et al., 2013; Zhu et al., 2014; Kumar et al., 2015; Singh et al., 2016; Singh et al., 2018; He et al., 2020; Roy et al., 2021; Gahtyari et al., 2021 and Kaur et al., 2021). Similarly, about a dozen GWA studies (ten studies) were also conducted for spot blotch (Adhikari et al., 2012; Gurung et al., 2014; Ahirwar et al., 2018; Ayana et al., 2018; Jamil et al., 2018; Juliana et al., 2019; Bainsla et al., 2020; Tomar et al., 2021; Juliana et al., 2022; Lozano-Ramirez et al., 2022). The present study is yet another attempt, adding 36 novel MTAs to the ever-growing list of markers associated with resistance against spot blotch. Some of these are recommended for use in MAS (see later).

At least a dozen models are now available showing significant improvement in GWAS. In the recent past several genome wide association studies have been conducted, where several models have been used and the results compared (Ayana et al., 2018; Ward et al., 2019; Chaurasia et al., 2020; Alemu et al., 2021; Malik et al., 2021a; Sandhu et al., 2021; Soumya et al., 2021). In this study, we selected five models for identification of main effect MTAs and PLINK for epistatic interactions. The merits and demerits of these models have been widely discussed (Gupta et al., 2014, Gupta et al., 2019; Kaler et al., 2020). Among these models, till recently, FarmCPU was a preferred model since it involves the use of Fixed Effect Model (FEM) and a Random Effect Model (REM) iteratively, and thus eliminates confounding problems arising due to kinship, population structure, multiple testing. However, FarmCPU is a model, which is based on unrealistic assumption that quantitative trait nucleotides (QTNs) are evenly distributed throughout the genome. BLINK approximates the maximum likelihood using Bayesian Information Content (BIC) in a fixed-effect model to eliminate the computational burden. In BLINK, REM is replaced with FEM to eliminate the requirement that QTNs are evenly distributed throughout the genome, which further improved the statistical power over FarmCPU, in addition to reduced computing time, so that in BLINK, the computational time is reduced from approximately one week in FarmCPU (Huang et al., 2018) to three hours in BLINK. The detailed difference among models is discussed by Gupta et al. (2019).

The single locus single trait analysis (used in CMLM and SUPER) has several limitations (Gupta et al., 2014). These two models were used in the present study mainly for the purpose of comparing their results with the three other improved multi-locus models that were used in parallel in this study. These new approaches included MLMM, FarmCPU and BLINK (Segura et al., 2012; Liu et al., 2016 and Huang et al., 2018). These models were also used in earlier studies, although BLINK was only sparingly used (Ayana et al., 2018; Bainsala et al., 2020; and Tomar et al., 2021). These five approaches (two for single locus; three for multi-locus) for GWAS used in the present study take into consideration the genetic background and epistatic interaction.

Epistatic interactions are often ignored in GWAS, although recently studied for the following traits: (1) flowering time (Reif et al., 2011; Langer et al., 2014), (2) stem rust resistance (Yu et al., 2011), (3) agronomic traits (Sehgal et al., 2017), (iv) yield related traits, micronutrients, and grain morphology (Jaiswal et al., 2016; Kumar et al., 2018; Malik et al., 2021a, Malik et al., 2022). Epistatic interactions were also detected through interval mapping (Li et al., 2011; Xu et al., 2012; Rouse et al., 2014; Boeven et al., 2020). However, epistatic MTAs have been sparingly used in MAS for crop improvement (Kao et al., 1999; Reif et al., 2011; Langer et al., 2014; Jaiswal et al., 2016; Sehgal et al., 2017; Kumar et al., 2018). We believe that all epistatic QTLs and MTAs, including those detected in this study, should be examined for their possible use in MAS.

### MTAs for MAS

Among the MTAs identified in this study, 13 MTAs suitable for marker-assisted selection (MAS) were initially selected using the following criteria: (1) lowest P-value, (2) identified by more than one models, (3) identified in more than one environment, (4) reported in earlier studies. These 13 MTAs included five MTAs for AUDPC, two MTAs for IP, six MTAs for LN (**Table 4**). Surprisingly, no multi-trait MTA was detected for more than one trait. Therefore, we need to consider the relative importance of three traits. Due to proven value of AUDPC in majority of past studies on genetics and breeding for SB, we like to recommend only five MTAs for AUPDC and ignore those for IP and LN. Since epistatic QTLs are also important, we like to add two MTAs only for AUDPC (**Table 3**) involving epistatic interaction. In this manner, we recommend that seven MTAs including five main effect MTAs and two epistatic interactions (four markers) should be examined for their use in MAS (or preferably marker assisted recurrent selection).

**Table 4:** A summary of most important MTAs common in four GWAS models (CMLM, MLMM, FarmCPU & BLINK; MTAs with SUPER not included).

Marker: SNP	Chr*	Pos*. of SNP tag (bp)	P. value	Description
AUDPC				
M1654: C/A	2B	797177741-797377809	4.09E-04	E3, E4
M4359: A/T	6B	22081AU357-22081425	6.66E-04	E4, B
M9772: A/G	3A	659038152-659038220	1.16E-04	E3, E4, B
M10783: G/A	7D	490344136-490344204	2.01E-04	E2, B
M8330**^#^**: A/C	2A	700374249-700374317	9.51E-04	QSb.bhu-2A
IP				
M4648: G/T	7A	94143005-94143073	5.89E-05	E3, E4
M11765: T/C	2A	107180183-107180251	3.55E-04	E4, B
LN				
M2771: A/C	4A	16997957-16998025	1.18E-04	E3, E4
M3928: G/C	2B	602182720-602182788	2.12E-04	E3, E4
M5313: T/C	2A	485122901-485122967	4.94E-04	E3, E4
M6140: T/C	5B	522912127-522912195	5.34E-04	E3, E4
M6730: T/C	1A	20893697-20893765	5.11E-04	E3, E4
M11585: G/A	7A	66298367-66298435	5.01E-04	E3, E4

*Chr, Chromosome; Pos, Position; bp, base pair **^#^** Lying within QTL interval of QSb.bhu-2A (Kumar et al., 2010).

### Candidate genes

Among the candidate genes identified in this study, genes encoding proteins with NBS-LRR domain superfamily are the most important, since these are the most common disease resistance genes (Lee and Yeom, 2015; Dubey and Singh, 2018; Tomar et al., 2021). Nine CGs (**Supplementary Table 7**) identified are known to be involved in defense response of wheat to *Puccinia triticina* causing leaf rust (Wang et al., 2019), *Zymoseptoria tritici* causing septoria tritici blotch (STB) and some other fungal diseases (He et al., 2018). The genes encoding proteins with F-box family are also known to mediate responses to biotic (Kim and Delaney, 2002) and abiotic stresses (Calderon-Villalobos et al., 2007); these genes are also known to control leaf senescence (Woo et al., 2001), stay-green trait (Tomar et al., 2021) and leaf blight resistance (Joshi et al., 2007a; Rosyara et al., 2008). Three CGs were associated with the genomic region, which encode proteins with Zinc finger domain and are known to take part in several traits including the following: (i) ABA/gibberellin stress response (Lin et al., 2011); (ii) seed germination (Kim et al., 2008); (iii) pathogen-associated molecular pattern-triggered immune (PTI) responses (Maldonado-Bonilla et al., 2013); (iv) salt stress response (Sun et al., 2007).

## Data Availability

The original contributions presented in the study are included in the article/**Supplementary Material**. Further inquiries can be directed to the corresponding author.

## Conclusions

The MTAs for three SB resistance traits i.e., AUDPC, IP and LN identified in the present study may be useful for MAS in breeding programs involving improvement in spot blotch resistance. These MTAs may be validated for use in MAS and may also be used for post-GWAS or joint linkage and association mapping (JLAM; Gupta et al., 2019). The information of the CGs may also be useful for the development of CG-based functional markers. The CGs identified in the present study may also be used for CG based association mapping and functional genomics in future research.

## Web links

^1^ Wheat QTL database (http://wheatqtldb.net/)

^2^ PLINK (https://zzz.bwh.harvard.edu/plink/index.shtml)

^3^ Ensembl database (http://www.ensembl.org/info/docs/tools/vep/index.html)

^4^ IWGSC (http://www.wheatgenome.org)

## Supplementary material

Supplementary Figure 1 Manhattan and Q-Q plots for AUDPC in E1, for all five models.

Supplementary Figure 2 Manhattan and Q-Q plots for AUDPC in E2, for all five models.

Supplementary Figure 3 Manhattan and Q-Q plots for AUDPC in E3, for all five models.

Supplementary Figure 4 Manhattan and Q-Q plots for AUDPC in E4, for all five models.

Supplementary Figure 5 Manhattan and Q-Q plots for IP in E1, for all five models.

Supplementary Figure 6 Manhattan and Q-Q plots for IP in E2, for all five models.

Supplementary Figure 7 Manhattan and Q-Q plots for IP in E3, for all five models.

Supplementary Figure 8 Manhattan and Q-Q plots for IP in E4, for all five models.

Supplementary Figure 9 Manhattan and Q-Q plots for LN in E1, for all five models.

Supplementary Figure 10 Manhattan and Q-Q plots for LN in E2, for all five models.

Supplementary Figure 11 Manhattan and Q-Q plots for LN in E3, for all five models

Supplementary Figure 12 Manhattan and Q-Q plots for LN in E4, for all five models.
