# Supplementary figures and images for: Genetics of spot blotch resistance in bread wheat (Triticum aestivum L.) using five models for GWAS
Source: Front Plant Sci. 2023 Jan 18;13:1036064. doi: 10.3389/fpls.2022.1036064 (PMC9891466; doi:10.3389/fpls.2022.1036064)

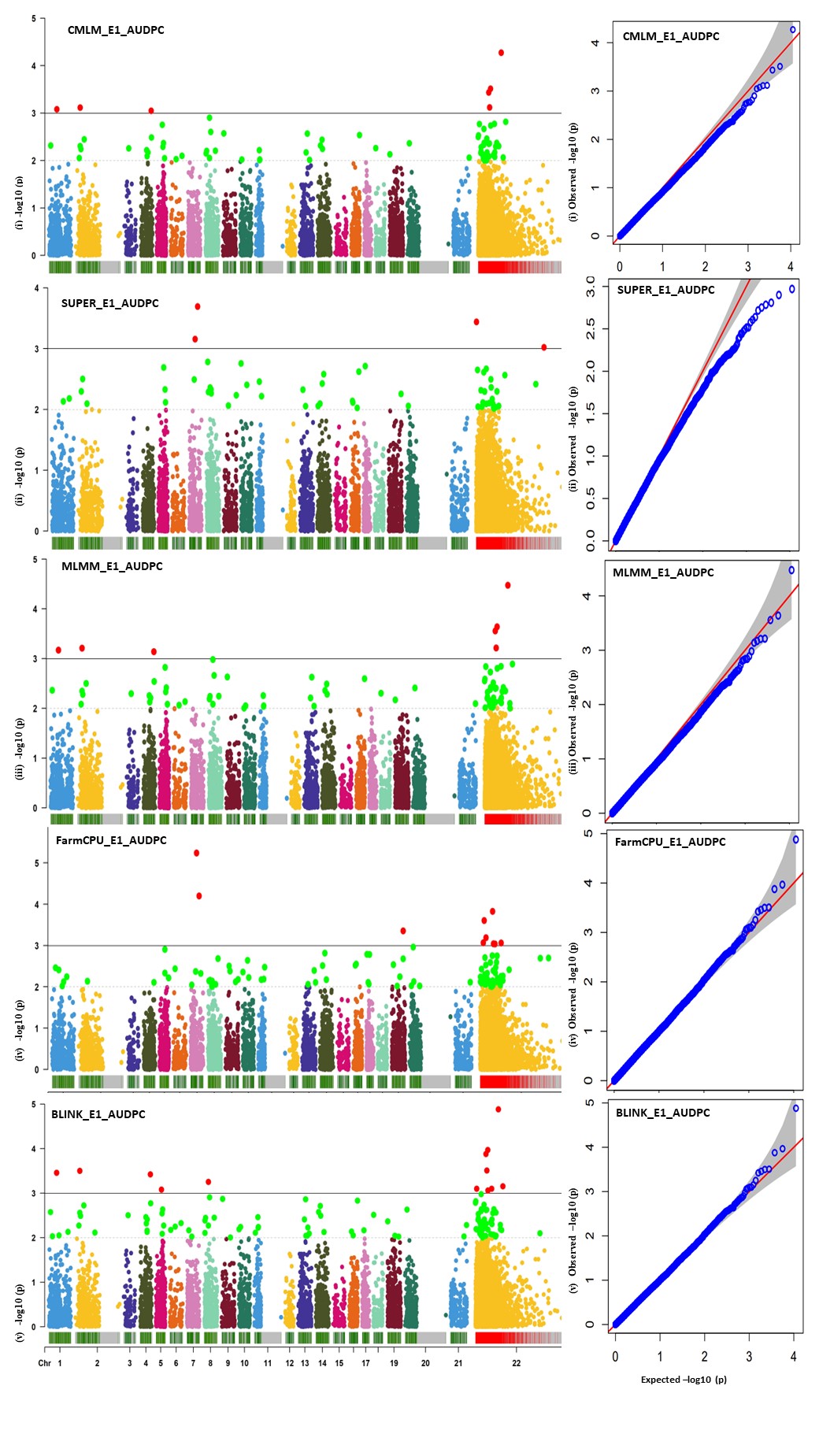

Supplement: Supplementary Figure 1 — Manhattan and Q-Q plots for AUDPC in E1, for all five models. [file Image_1.jpeg]

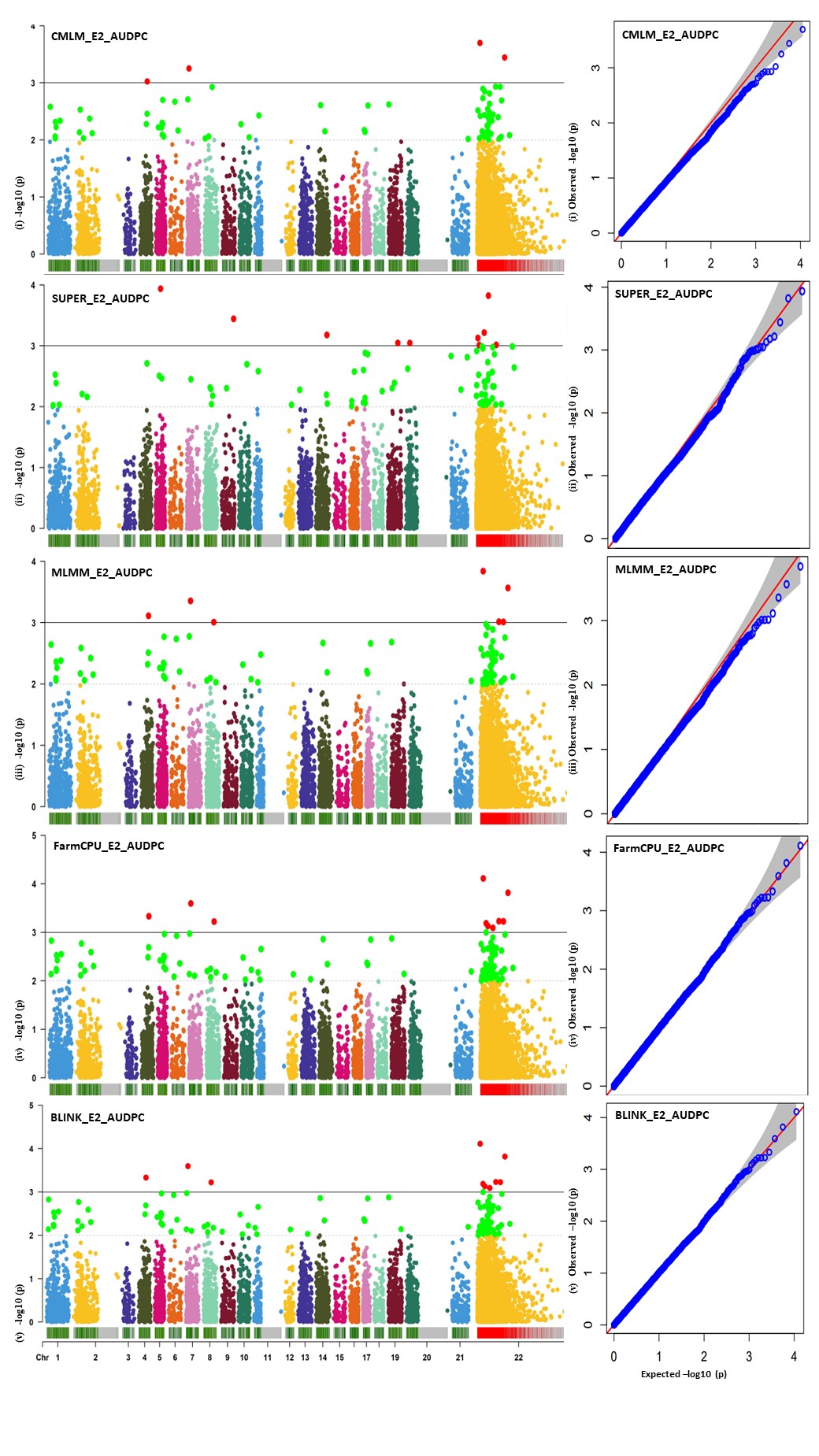

Supplement: Supplementary Figure 2 — Manhattan and Q-Q plots for AUDPC in E2, for all five models. [file Image_2.jpeg]

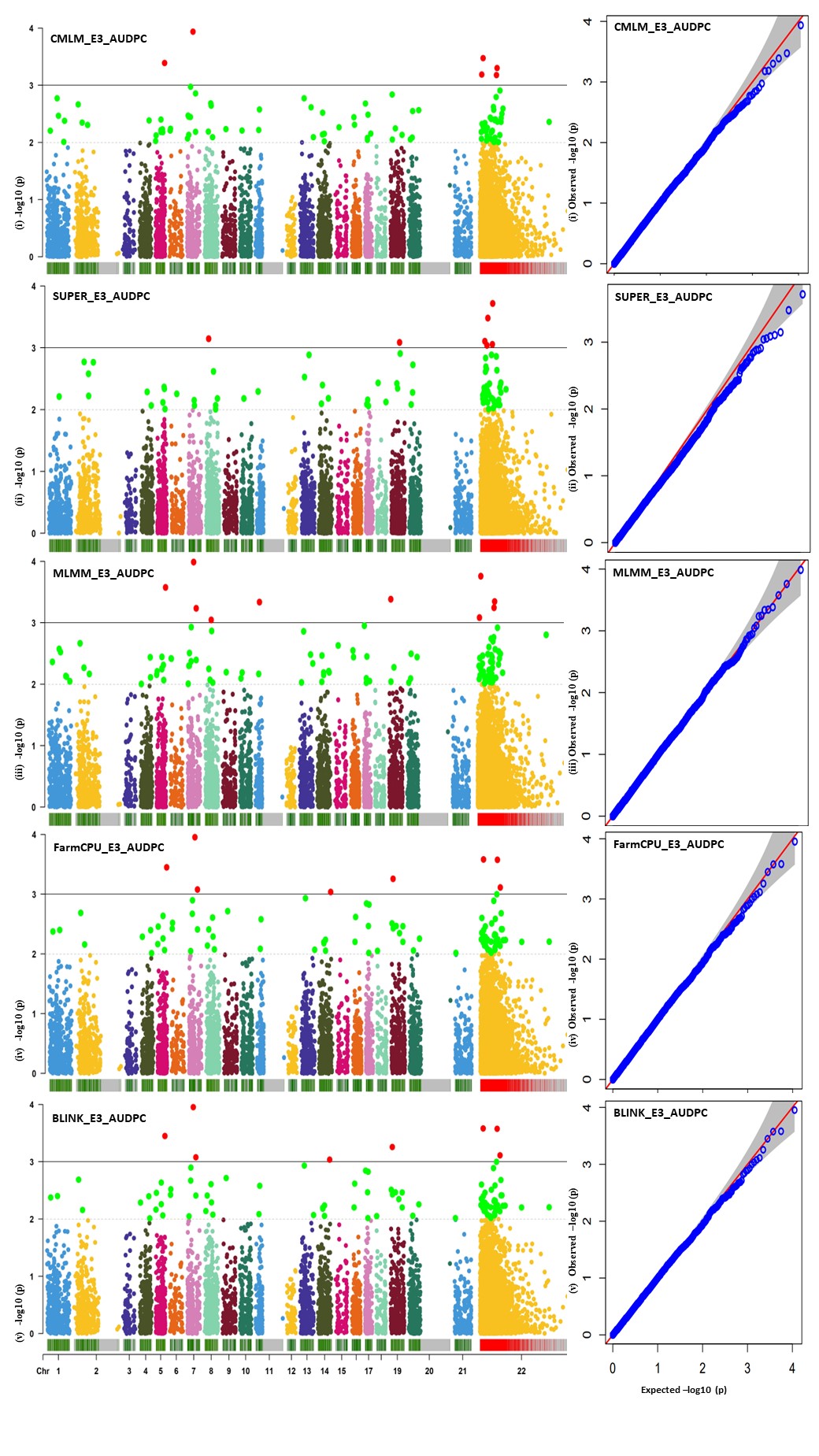

Supplement: Supplementary Figure 3 — Manhattan and Q-Q plots for AUDPC in E3, for all five models. [file Image_3.jpeg]

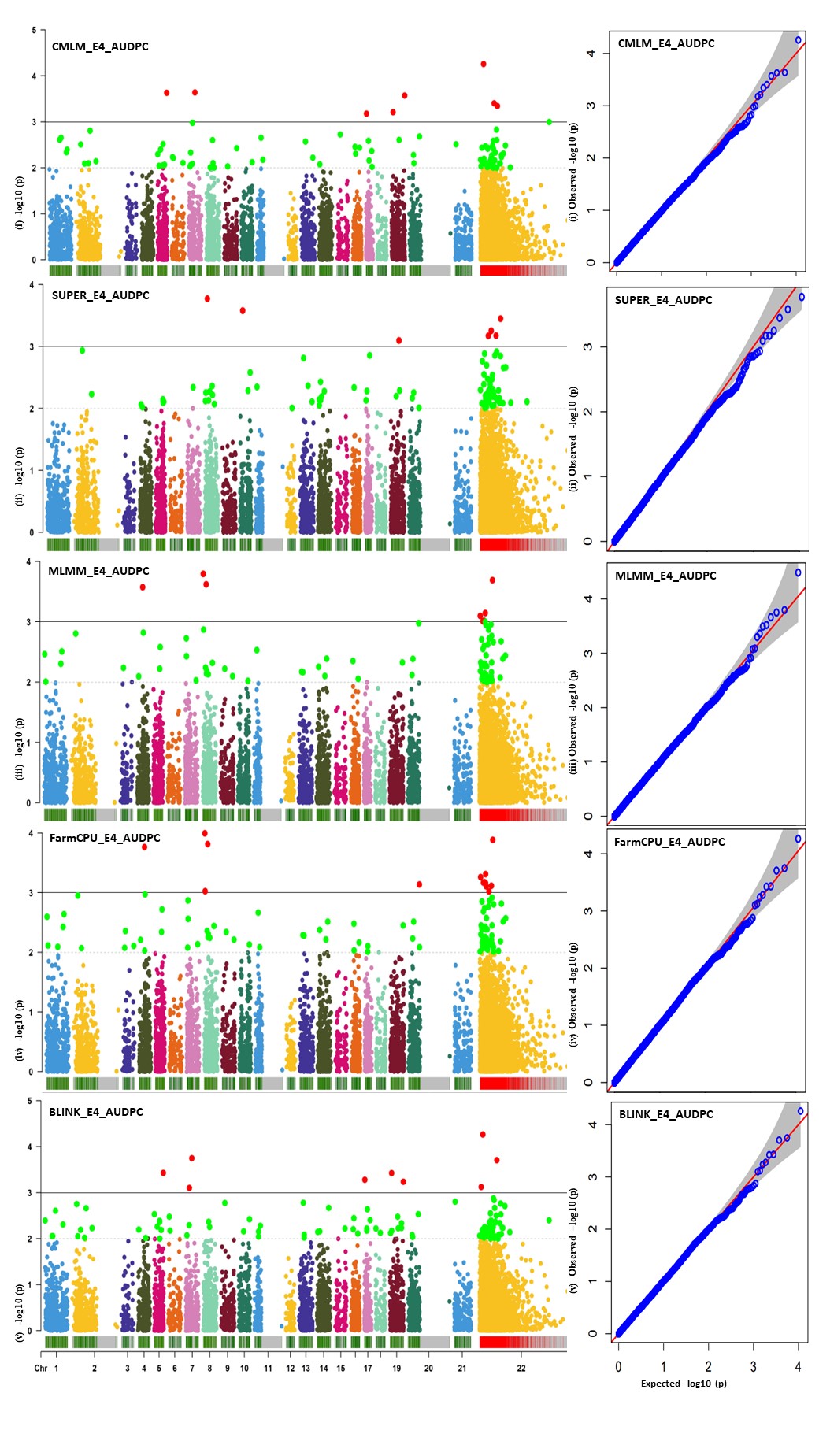

Supplement: Supplementary Figure 4 — Manhattan and Q-Q plots for AUDPC in E4, for all five models. [file Image_4.jpeg]

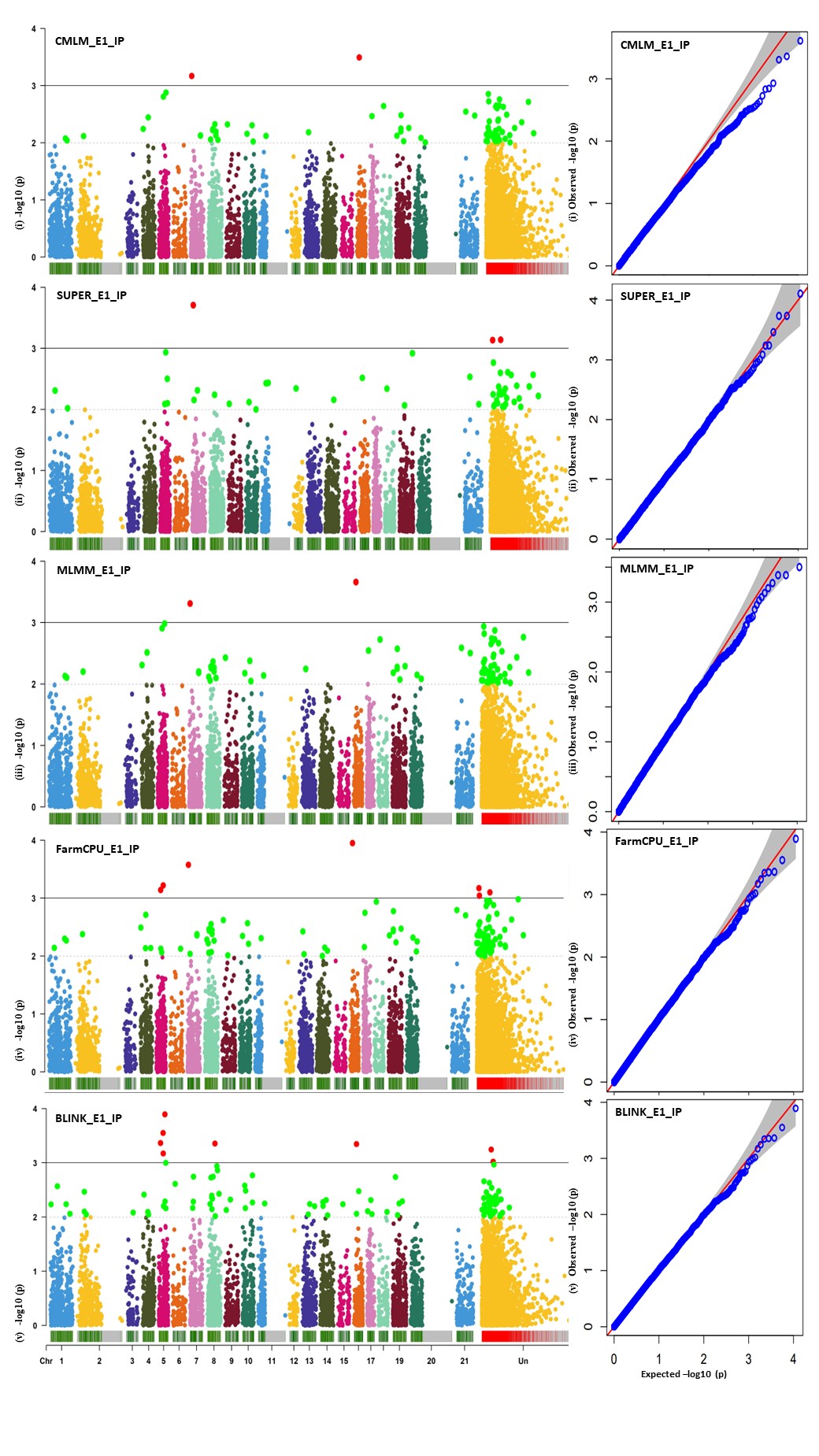

Supplement: Supplementary Figure 5 — Manhattan and Q-Q plots for IP in E1, for all five models. [file Image_5.jpeg]

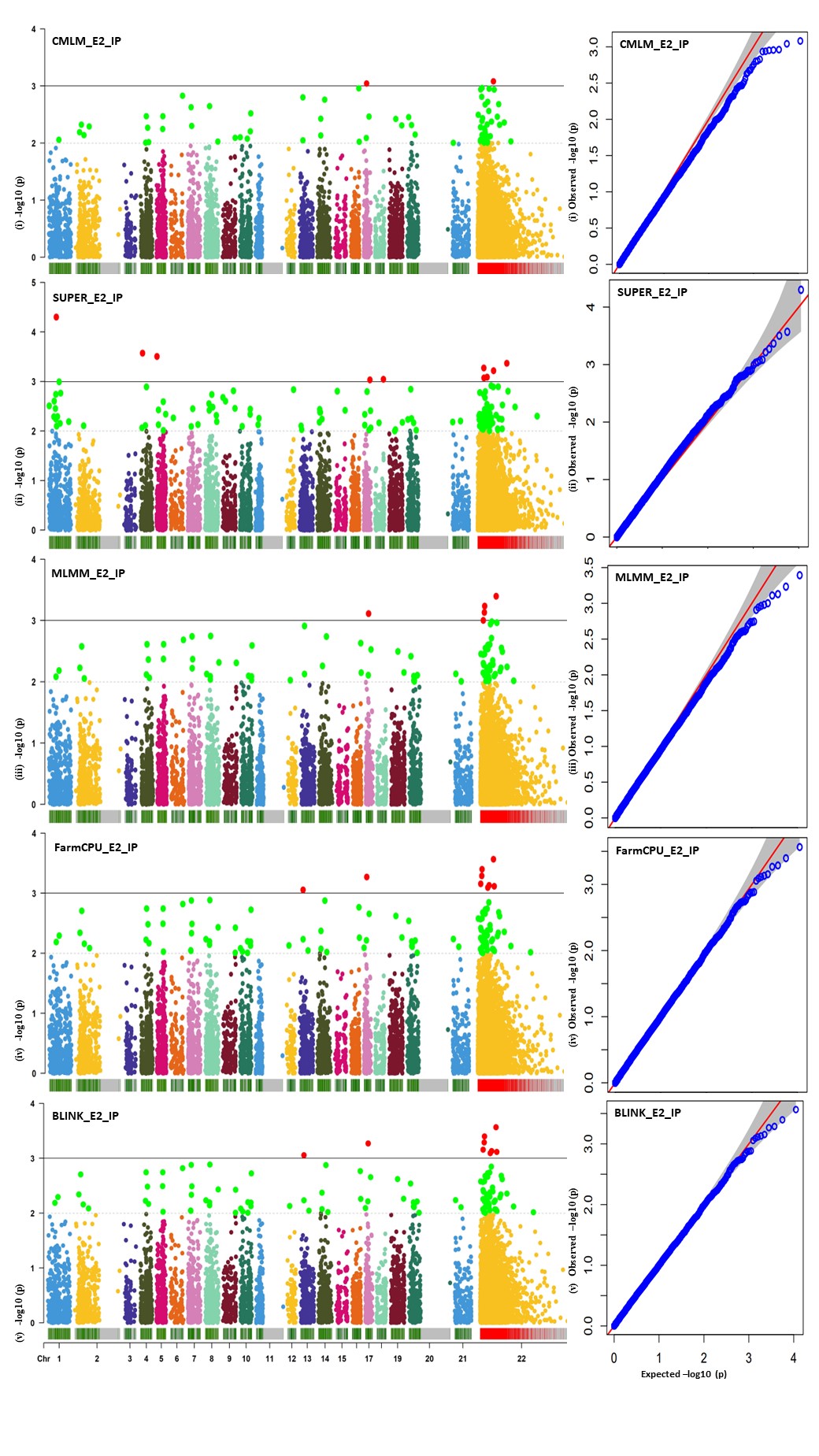

Supplement: Supplementary Figure 6 — Manhattan and Q-Q plots for IP in E2, for all five models. [file Image_6.jpeg]

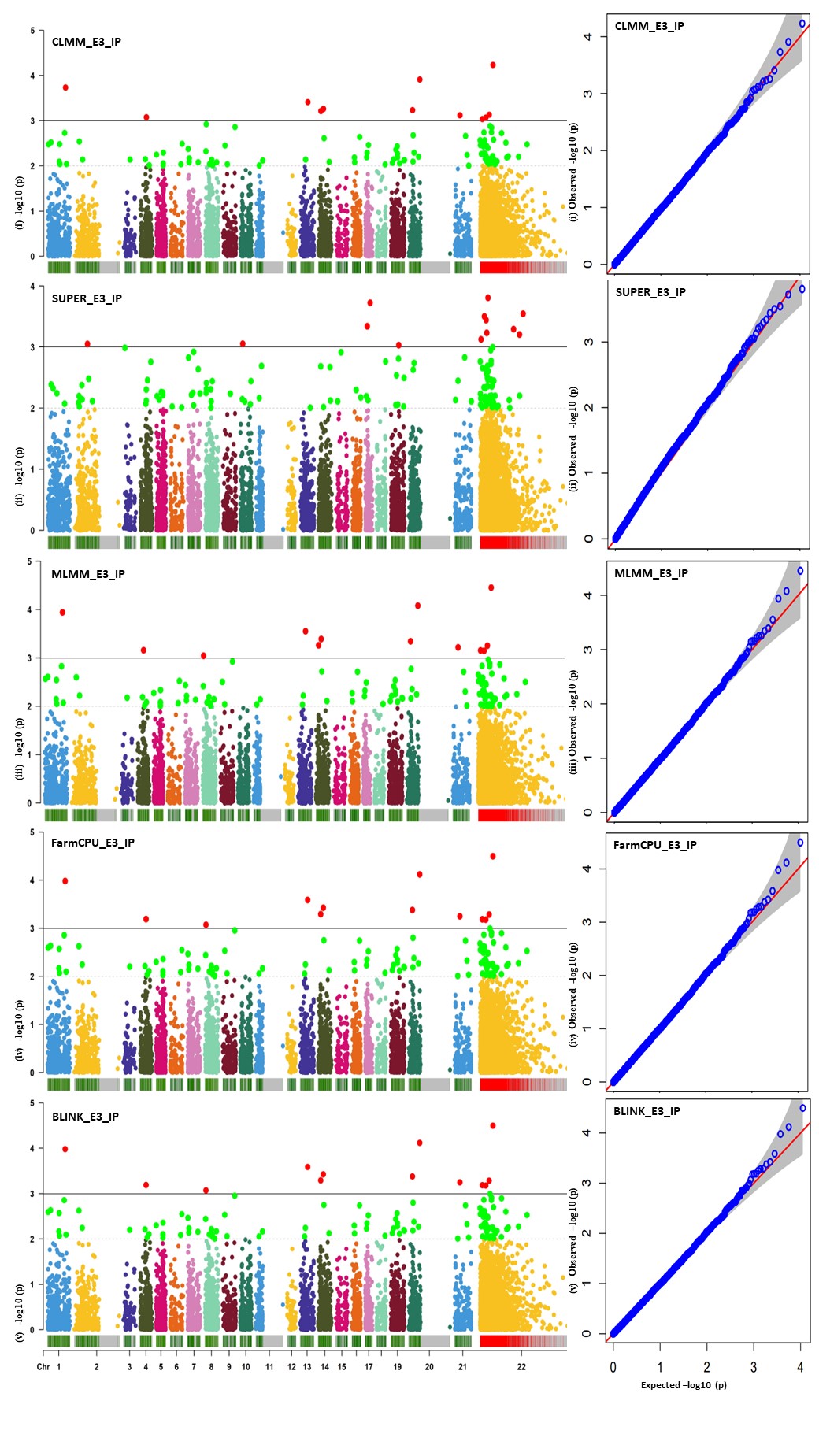

Supplement: Supplementary Figure 7 — Manhattan and Q-Q plots for IP in E3, for all five models. [file Image_7.jpeg]

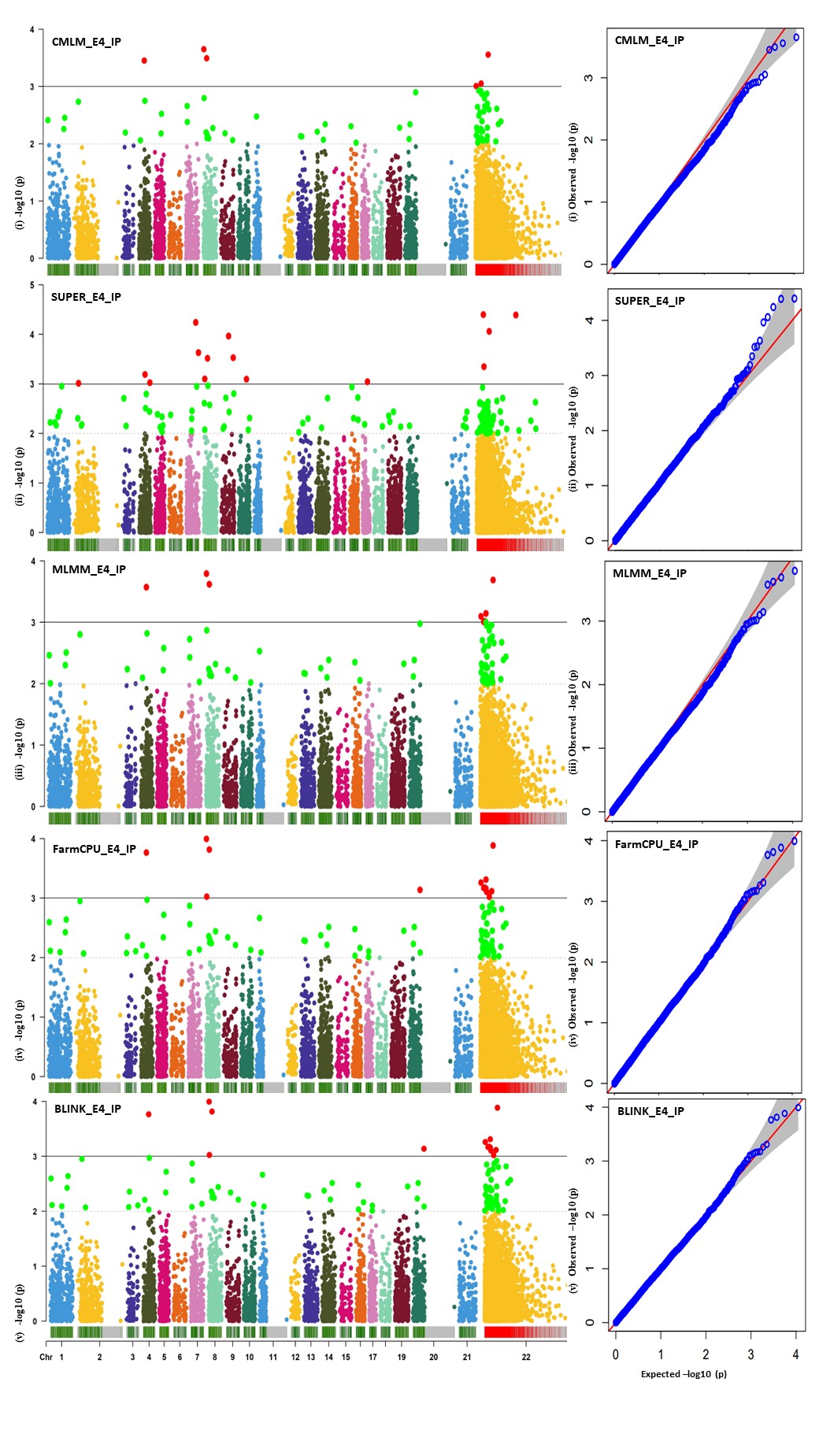

Supplement: Supplementary Figure 8 — Manhattan and Q-Q plots for IP in E4, for all five models. [file Image_8.jpeg]

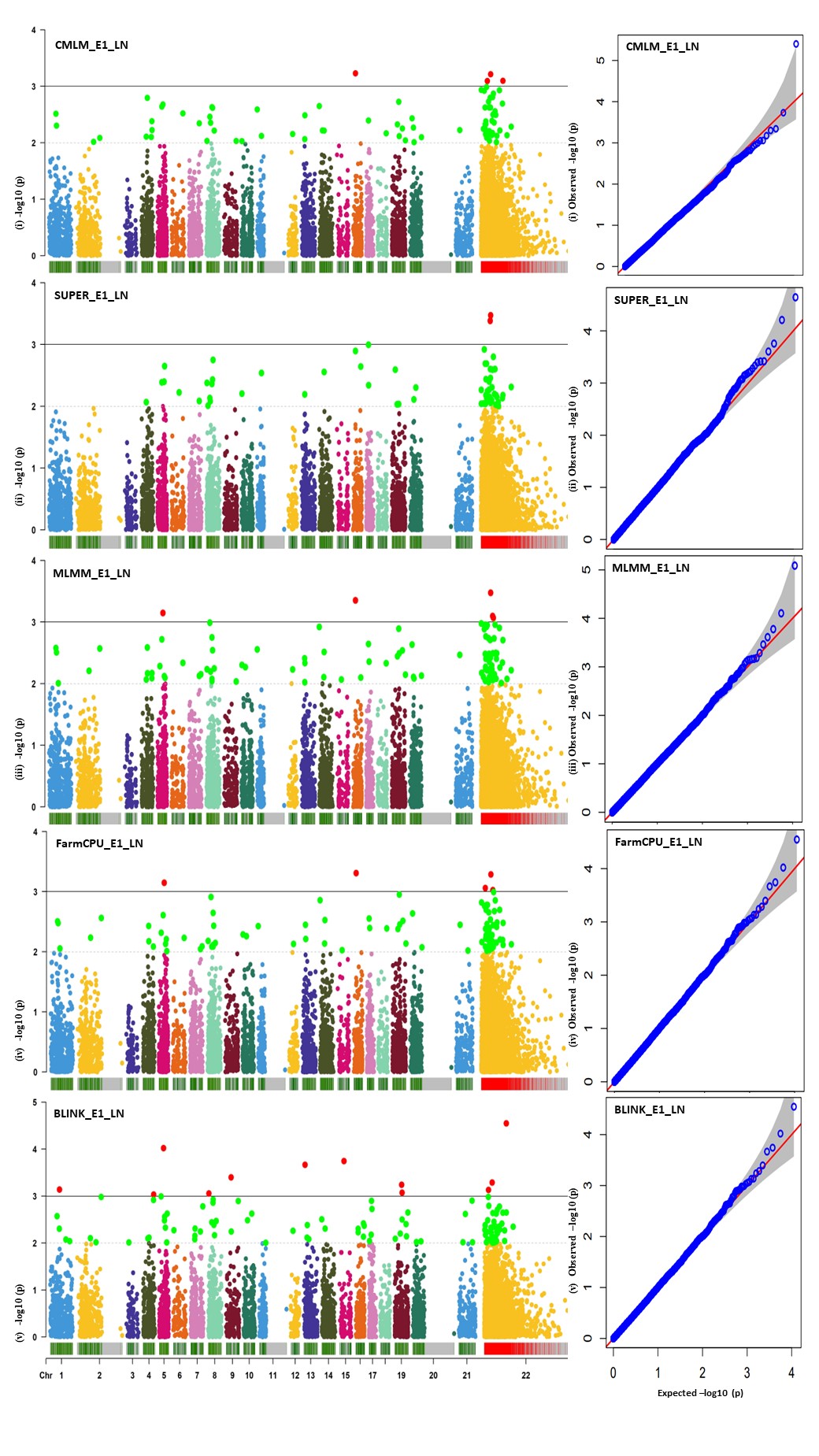

Supplement: Supplementary Figure 9 — Manhattan and Q-Q plots for LN in E1, for all five models. [file Image_9.jpeg]

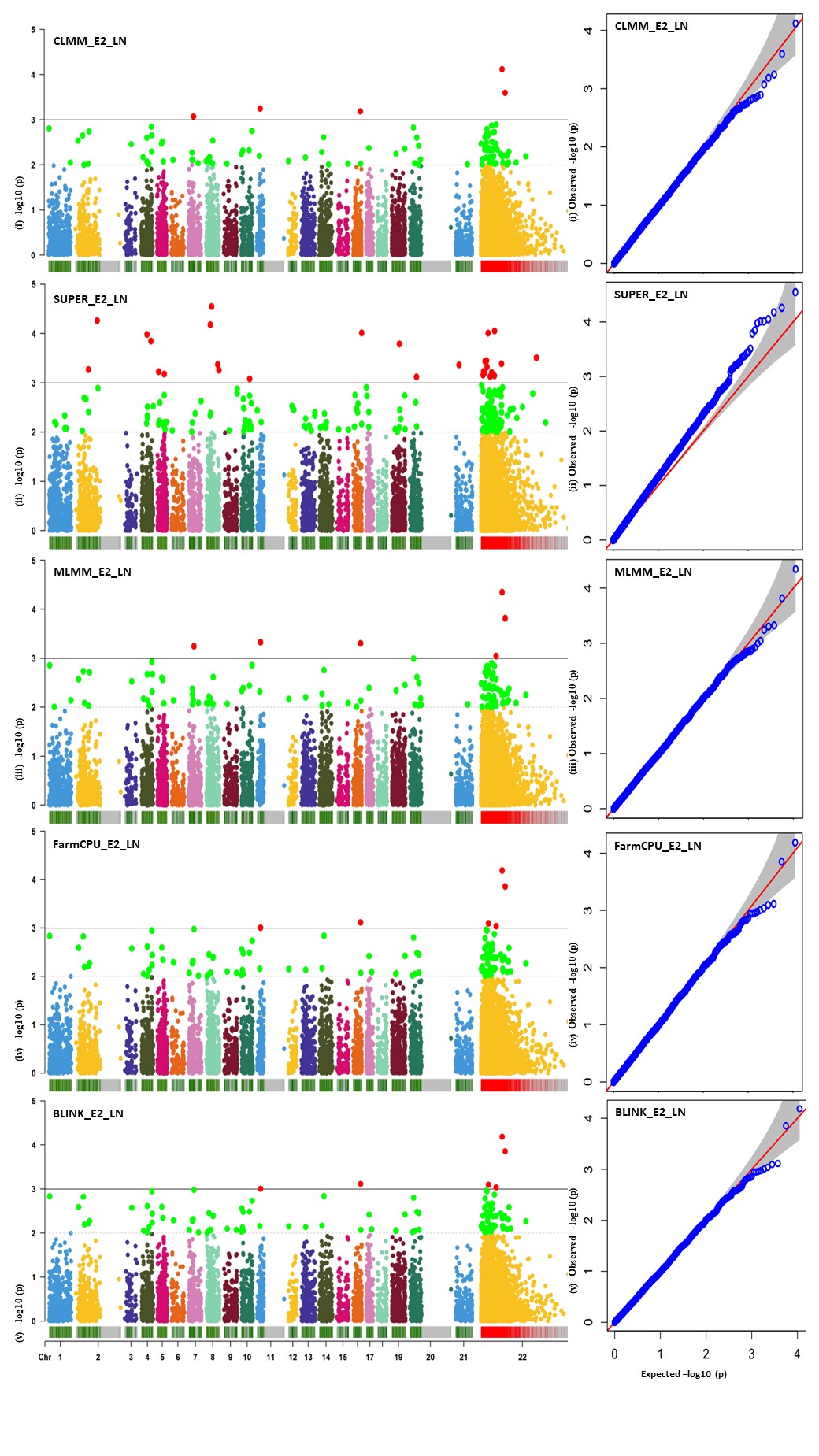

Supplement: Supplementary Figure 10 — Manhattan and Q-Q plots for LN in E2, for all five models. [file Image_10.jpeg]

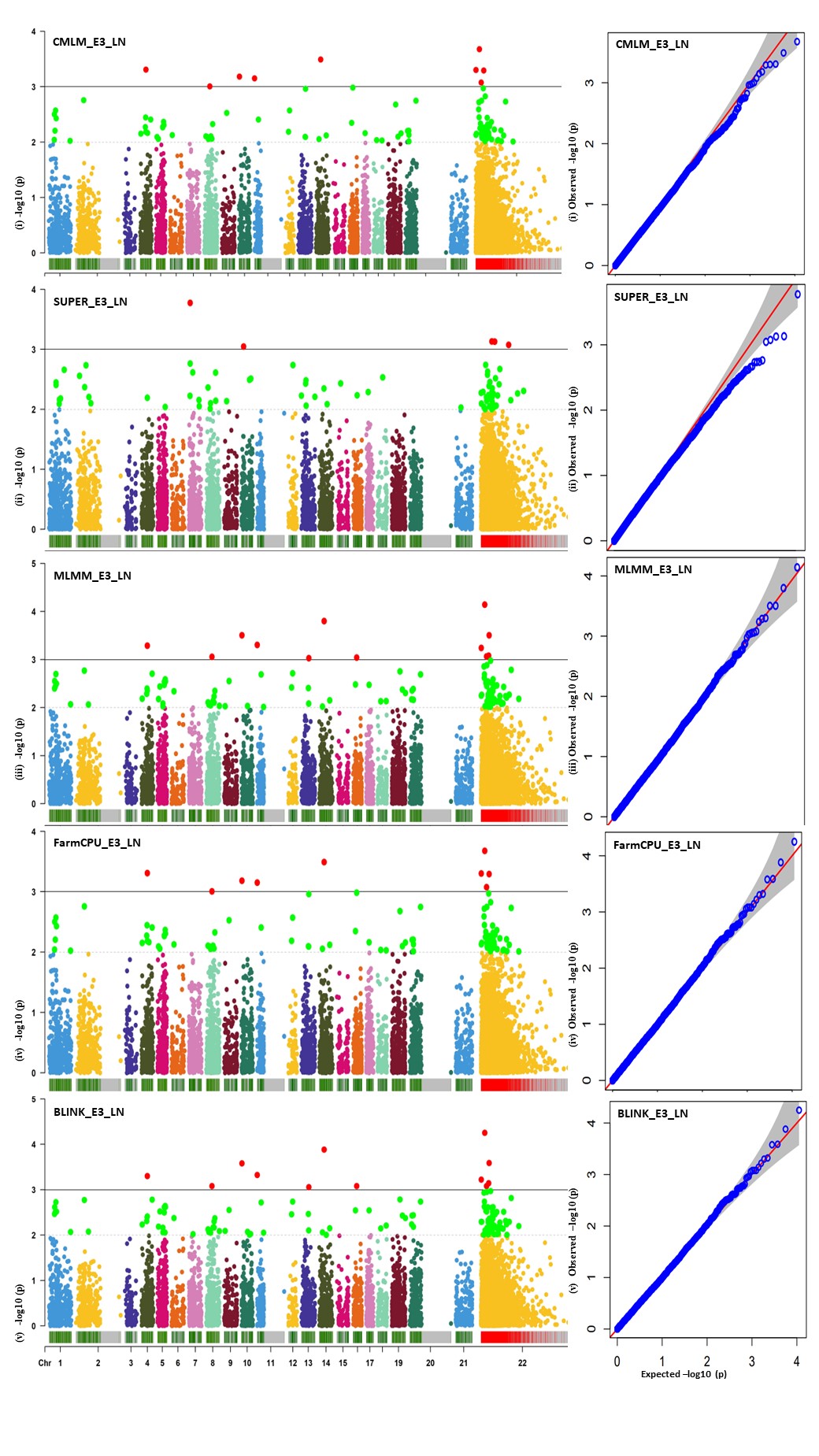

Supplement: Supplementary Figure 11 — Manhattan and Q-Q plots for LN in E3, for all five models [file Image_11.jpeg]

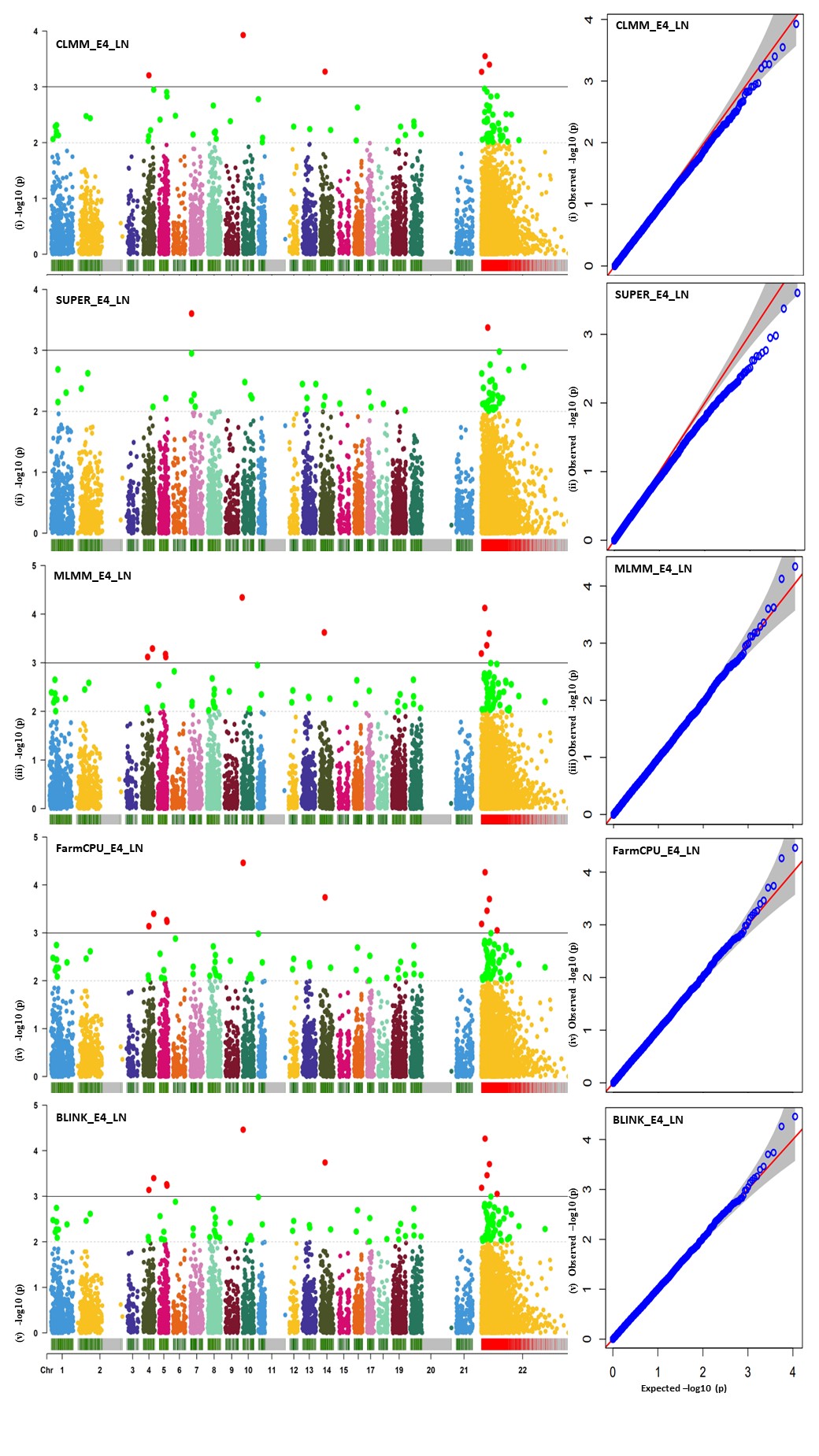

Supplement: Supplementary Figure 12 — Manhattan and Q-Q plots for LN in E4, for all five models. [file Image_12.jpeg]
